# A Live Single-Cycle RSV Vaccine with Immune Responses Focused on Prefusion F and the Central Conserved Region of Attachment Protein G

**DOI:** 10.3390/v18091013

**Published:** 2026-09-14

**Authors:** Jeeviya Murugesan, Patil Basavaraju Nanjegowdu, Pramila Lamichhane, Megolhubino Terhüja, Valerie McElliott, Antonius G. P. Oomens

**Affiliations:** 1 Department of Veterinary Pathobiology, College of Veterinary Medicine, Oklahoma State University, Stillwater, OK 74078, USA; jeeviya.murugesan@okstate.edu (J.M.);; 2 Laboratory of Infectious Diseases, National Institute of Allergy and Infectious Diseases, National Institutes of Health, Bethesda, MD 20892, USA; 3 Neovaxsyn, Inc., Ames, IA 50014, USA

**Keywords:** respiratory syncytial virus, RSV, vaccine, attachment protein, G protein, fusion protein, prefusion F, preF

## Abstract

*Respiratory syncytial virus* (RSV) is a major cause of severe lower respiratory tract disease in infants and children, and a pediatric vaccine is not available. Among various approaches, live vaccines are attractive as they induce broad multi-antigenic responses and have never been associated with vaccine-enhanced disease in RSV-naïve children. We previously reported a live prefusion F (preF) expressing single-cycle RSV vaccine, which was deficient in cell–cell transmission and yet induced protective responses in mice. To improve efficacy, we focused the anti-attachment protein (G) response on the relatively conserved G central region (GCR). Anti-GCR antibodies were shown to cross-protect against different RSV strains, and the GCR contains a receptor-binding domain. Focusing on the GCR was done by replacing the G open reading frame with that of a hybrid protein displaying the GCR, while also retaining stabilized preF expression. The resulting virus, RSV-preF-GCR, was readily produced in complementing cells, expressed preF and GCR at the infected cell surface, and maintained its single-cycle phenotype. In mice, intranasal prime-boost vaccination induced serum anti-preF and moderate levels of anti-G antibodies, which were specific for the GCR. RSV-preF-GCR also significantly reduced lung pathology after challenge with wild-type RSV. However, relative to a vaccine expressing full-length G, in vitro neutralization by RSV-preF-GCR-induced antibodies was poor, and lung IgA responses were unexpectedly low. Thus, RSV-preF-GCR is promising in uniquely combining single-cycle replication and induction of both anti-preF and anti-GCR immunity but likely requires efficacy improvements to enhance anti-G antibody levels and induce mucosal immunity.

## 1. Introduction

*Respiratory syncytial virus* (RSV) is a common worldwide respiratory pathogen that can lead to severe bronchiolitis and pneumonia in newborns, children, the elderly, and individuals with a compromised immune system [1,2,3,4,5,6]. In the United States, RSV is the most common cause of respiratory tract infection and hospitalization [7]. In the last few years, significant progress has been made toward anti-RSV modalities. Currently, the FDA has approved three RSV vaccines: Arexvy, Abrysvo (recombinant protein vaccines), and mRESVIA (mRNA vaccine). These vaccines target the fusion protein (F), one of the two major RSV antigens, and are licensed for use in adults aged 60 and older. One of these, Abrysvo, is also approved as a maternal vaccine to protect newborns via transplacentally acquired antibodies (Abs) and is targeted to pregnant women between 32 and 34 weeks of gestation [8]. Additionally, Nirsevimab and Clesrovimab, long-acting monoclonal Abs administered as single doses, have been authorized for prophylactic use in term and late preterm newborns [9,10,11,12]. However, a pediatric vaccine for RSV is not yet available.

In 1967, a formalin-inactivated RSV (FI-RSV) vaccine was developed and tested in clinical trials in young children. Rather than being protected, 80% of vaccinated children were hospitalized when they encountered RSV, due to a phenomenon called vaccine-enhanced disease (VED), and two vaccinees died following natural RSV infection [13]. The causes of VED are incompletely understood but involve, among others, a Th2-biased immune response [14,15]. Later studies found that, in contrast to subunit and inactivated vaccines, live vaccines are not associated with VED and have been proven safe in RSV-naïve recipients [16,17,18]. Live vaccines are attractive in other respects as well. They induce both humoral and cellular responses, which include mucosal immunity if applied intranasally (IN). As such, they can induce broadly protective immunity that also avoids dependence on a singular antigen and reduces the likelihood of escape variants in the long term. Thus, live vaccines are an attractive approach to develop a safe and efficacious vaccine to prevent RSV infection and disease in infants and children.

Live vaccines do, however, require stringent safety measures. In an effort to address vaccine safety and, in the long term, balance safety and efficacy, we previously designed self-limited or ‘single-cycle’ vaccines. These vaccines lack an essential component or function, which is provided in trans in production cell lines during batch preparation. When used to vaccinate animals or infect regular cell types, these viruses are infectious and transcribe and replicate at high levels but fail to form infectious progeny and transmit to other cells [19,20,21,22]. The lack of cell–cell transmission adds to the safety profile of single-cycle vaccines. One of our previously reported single-cycle viruses (RSV-preF-^∆CT^) expressed a DS-Cav1-based prefusion F protein (preF) and induced a higher proportion of preF:postF Abs, as well as robust T cell responses [20]. This vaccine also protected animals from challenge virus replication in the lung, and from virus-induced lung pathology.

Although single-cycle viruses show promise as vaccine candidates and displayed good efficacy/safety profiles in mice, we anticipate that higher efficacy will be needed to be effective in humans. One approach to improve efficacy focuses on the second major viral glycoprotein, attachment protein G. In contrast to F, the G protein is a type II transmembrane protein and highly divergent between subgroups A and B. The divergence is due to its mucin-like flanking regions, whereas the central region of the protein (corresponding approximately to amino acids 160–205) is relatively conserved [23,24]. This region was previously termed the G-CCR (central conserved region). The G-CCR contains a portion of a linear heparin-binding domain (HBD) which is believed to span amino acids 184–231 and helps facilitate viral entry in cultured cells via glycosaminoglycans [23,25,26,27]. It also harbors an important receptor-binding domain (RBD), the CX3C site [28,29]. G uses CX3C to bind to CX3CR1 on airway ciliated epithelial cells [28,29] and, as such, mediates in vivo entry. Binding to CX3CR1 can also modulate host immune responses and exacerbate inflammation [30]. Importantly, anti-G-CCR Abs were shown to cross-protect against multiple RSV strains, to have anti-inflammatory effects, and to shift the response from Th2 to Th1, underscoring the G-CCR as a highly relevant target for RSV vaccines and antiviral therapies [31,32,33,34,35,36].

We previously generated a hybrid RSV protein that consists of the F carboxy-terminal domain (Fstem) with the G central region grafted onto it [37]. Different lengths and variants were tested to arrive at Fstem/GCR, a protein that contains G residues 137–211 and was validated for display at high levels at the cell surface and for recognition by known anti-G Abs [37]. This construct was initially made to generate virus-like particles but can also be integrated into a live vaccine.

In this project, we generated a novel vaccine termed RSV-preF-GCR, in which we replaced the G open reading frame (ORF) of a single-cycle preF virus with one encoding Fstem/GCR, in an effort to focus the anti-G response on the central region of G. If successful, RSV-preF-GCR could gain efficacy in humans by targeting the immune response to the central region, which harbors an RBD, and by gaining cross-protective ability as the central region is better conserved [38,39]. In addition, focusing the vaccine response on the GCR will likely induce fewer non-neutralizing Abs after infection by strains with high variability in the G flanking regions. Since non-neutralizing Abs have been implicated in VED [40,41], raising the proportion of neutralizing anti-G Abs may therefore provide additional safety. Along with a control virus expressing a full-length G protein, RSV-preF-GCR was tested in vitro and in vivo, and advantages and challenges associated with its vaccine potential are discussed.

## 2. Materials and Methods

### 2.1. Cells and Abs

Vero76 (Vero) and HEp-2 cells (American Type Culture Collection) were maintained in advanced Dulbecco’s Modified Eagle medium (DMEM) supplemented with 4% fetal bovine serum, 50 units/mL penicillin, 50 µg/mL streptomycin, and 2 mM glutamax (Invitrogen, Cat. No. 35050061). Vbac cells expressing chimeric glycoprotein GP^64/F^ (baculovirus GP64 protein with a partial cytoplasmic tail from RSV F protein) were previously generated [42] and were maintained in the same medium plus 0.2 mg/mL G418 every other passage. Monoclonal antibodies (MAb) D25 and Motavizumab were kindly provided by Jason McLellan (University of Texas at Austin, Austin, TX, USA). MAb L9 [43] was provided by Edward Walsh (University of Rochester School of Medicine, Rochester, NY, USA). Ab B023 (Anti-N Ab) was purchased from Bio-Rad (Hercules, CA, USA). Anti-FLAG Ab was acquired from Genscript (Piscataway, NJ, USA).

### 2.2. Construction of Viral cDNAs

A hybrid protein termed Fstem/GCR was previously reported [37]. In essence, using basic molecular techniques, the sequence encoding the ectodomain of F (amino acids 36–495) was replaced with one encoding G amino acids 137–211. This hybrid Fstem/GCR protein also has the final two F amino acids mutated to alanine [37] and the glycosylation site N500 mutated to threonine [44]. For this study, to avoid any premature cathepsin cleavage at leucine 208, this residue was mutated to alanine [27], and the product was named Fstem/GCR-208A. To move the GCR constructs into cDNA and generate single-cycle preF-based viruses, we started with RSV-preF. The latter is a previously described A2-based cDNA that expresses preF from the 6th genome position, a membrane-anchored full-length G protein (M48L mutation to ablate secreted G expression) from the 7th genome position, and GFP from the 8th genome position (see Section 3 for schematic). Note that in our previous work [20], RSV-preF was termed RSV-preF^∆CT^. To generate RSV-preF-G208A, leucine 208 was mutated to alanine to avoid premature cathepsin cleavage at leucine 208 [27]. To generate RSV-preF-GCR, the G ORF was excised from RSV-preF and replaced with an ORF encoding Fstem/GCR-208A, using remote-cutting restriction enzymes and conventional cloning techniques. All final constructs were sequence-verified.

### 2.3. Recovery of Viruses from cDNA and Production of Virus Stocks

Infectious viruses were recovered from cDNAs as previously described [19,20,21,45] with minor modifications. Briefly, BHK-21 cells stably expressing T7 polymerase were transfected with engineered RSV cDNA constructs using Lipofectamine 2000 (Invitrogen, Carlsbad, CA, USA) in Opti-MEM reduced-serum medium (Gibco, Waltham, MA, USA) according to the manufacturer’s instructions. Support plasmids were used containing internal ribosome entry sites and encoding the nucleoprotein (N), phosphoprotein (P), transcription elongation factor (M2-1), and polymerase (L), at 0.3, 0.35, 0.15, and 0.2 µg per well, respectively. In addition, 0.5 mg of a plasmid expressing GP^64/F^ (see Section 2.1) was included to enable assembly of infectious progeny virus in the transfected cells [42,46]. After incubation at 33 °C for 70 h, supernatants from transfected cells were transferred to Vbac cells and incubated for an additional 7 days at 33 °C. Prior to harvest, cell medium was replaced with advanced DMEM containing 7% sucrose and 40 mM HEPES, and cells were collected by scraping into the sucrose-containing medium. Collected suspensions were pipetted up/down vigorously for 30 strokes, and cell debris was removed by low-speed centrifugation. The resulting viral stocks were frozen at −80 °C and designated as low-passage seed stocks. All virus stocks used in subsequent experiments were generated directly from these seed stocks. For vaccine batch production, Vbac cells were infected with seed stocks and incubated at 33 °C for 7 to 8 days. Virus was harvested by scraping cells into medium, 50 strokes of vigorous pipetting, and removal of cell debris by low-speed centrifugation. Virus particles were then pelleted by ultracentrifugation at 40,000× *g* through a 25% sucrose cushion. Following centrifugation, virus pellets were washed with advanced DMEM, resuspended in advanced DMEM containing 40 mM HEPES and 100 mM MgSO_4_, flash-frozen in liquid nitrogen, and stored at −80 °C. Virus titers were determined by plaque assay, with plaques scored based on GFP expression at 3–5 days post-infection. The genomic integrity of engineered viruses was confirmed by bulk sequencing of modified genome areas after viral RNA purification from infected cells and reverse transcription (RT)-PCR amplification.

### 2.4. Cell ELISA

Cell ELISAs were performed on infected HEp-2 cells at 26 h post-infection as previously described with minor modifications [20]. To detect preF, G, and Fstem/GCR, primary Abs D25 (Ab specific for preF only) and L9 (anti-RSV-G Ab: recognizes both G and Fstem-GCR) [43] were incubated with unfixed cells (to maintain native conformation) for 2 h. Note that in results Section 3.5 specifically, the primary Abs were mouse Abs induced by vaccination with RSV-preF-G208A and RSV-preF-GCR. To detect the FLAG tag, an anti-FLAG Ab (Genscript, Piscataway, NJ, USA) was used. After washing away unbound Abs, cells were fixed for 5 min with freshly dissolved 4% paraformaldehyde. This was followed by incubation with a horseradish peroxidase (HRP)-conjugated secondary Ab and washing steps. 0.1% BSA was included in both Ab incubation steps to prevent non-specific signal. After the last wash step, cells were incubated in O-phenylenediamine dihydrochloride (Fisher Thermo Scientific, Waltham, MA, USA)-based ELISA substrate, and the reaction was stopped by adding 3M sulfuric acid. The optical density at 490 nm (OD_490_) was measured in a Versamax plate reader (Molecular Devices, San Jose, CA, USA). For normalization of infected cells, N protein was measured by permeabilizing paraformaldehyde-fixed cells with 0.2% triton, prior to incubation with anti-N Ab. Error bars indicate standard deviation from the mean of triplicate samples. Each experiment was carried out twice independently with similar results.

### 2.5. Western Blot Analysis

Semi-purified virus particles were pelleted at high g-force (21,000× *g*, 60 min) and resuspended and heated in Laemmli buffer. Equivalent PFUs of all viruses were electrophoresed by reducing 12% SDS-PAGE and transferred to Immobilon blots (Millipore Sigma, Burlington, MA, USA) using a semi-dry apparatus (Bio-Rad, Hercules, CA, USA). The blots were blocked with 5% non-fat milk powder in TBS-tween. Blocked membranes were washed and probed with primary Abs anti-F (Motavizumab), anti-M [45], or anti-G (L9) and incubated for 2 h. Membranes were then incubated with HRP-conjugated secondary Ab for 1 h. Blots were developed using enhanced chemiluminescence and scanned on an Amersham Imager 600 (GE Healthcare, Chicago, IL, USA). The experiment was carried out three times, and a representative blot is shown. Protein band intensities were quantified by densitometry using ImageJ software version 1.54 (NIH, Bethesda, MD, USA), and average ratios of F:M and G:M were calculated. Bars represent the mean of two individual blots.

### 2.6. Cell–Cell Transmission

Vero and Vbac cells were infected at 0.01 PFU per cell. Following infection, GFP fluorescence was evaluated on days 1, 5, and 9 post-infection to track virus dissemination between neighboring cells. Cells were fixed with freshly prepared 4% paraformaldehyde for 5 min, counterstained with Hoechst, and images were acquired using a BioTek Cytation 5 (Agilent, Winooski, VT, USA), with the exception of Vbac day 1 images, which were taken on a Nikon TE2000 inverted fluorescence microscope (Nikon Instruments Inc., Melville, NY, USA). All images were acquired at 100× magnification.

### 2.7. Mouse Vaccination and Challenge Studies

For vaccination, eight-week-old female BALB/c mice (*n* = 5 per group) were anesthetized by intraperitoneal injection of 100 mg/kg of body weight of ketamine and 5 mg/kg of xylazine. Mice received prime and boost IN vaccinations three weeks apart, each consisting of 0.5 million PFU in a 50 µL volume. The mock group received an equal volume of material identically prepared from uninfected Vbac cells. At 21 days post-boost, blood samples were collected for serum Ab studies. In another study, mice were immunized as above, and perfused lung samples were collected 15 days post-boost to measure lung IgA levels. Lungs were homogenized in PBS containing protease inhibitors using a precellys bead homogenizer (Bertin Technologies, Saint-Quentin-en-Yvelines, France) (2 × 20 s at 8500 rpm); debris was cleared by low-speed centrifugation, and the suspension was stored at −80 °C until further analysis.

For challenge studies, vaccinated mice were infected IN with 1 million PFU of RSV-K191R (wild-type RSV carrying the line19F mutation K191R, previously called A2/K191R) [21], four weeks after the boost. The mock-mock vaccinated group was challenged with mock-infected cell lysate. Five days post-challenge, mice were humanely euthanized, and lungs were collected for histopathological analysis. Lungs were gently inflated with 10% neutral-buffered formalin and immersed in the same fixative to ensure complete fixation. Fixed tissues were processed through graded alcohols and xylene, embedded in paraffin, sectioned at 4 mm, and stained with hematoxylin and eosin (H&E). Histopathological evaluation was performed in a blinded manner by an ACVP board-certified veterinary anatomic pathologist. Lung pathology was scored across five parameters: interstitial pneumonia (increased thickness of alveolar septa, leukocytes in the alveolar space), edema (extrusion of amorphous eosinophilic material into the airways and surrounding blood vessels, and/or presence of foamy macrophages), perivascular cuffing (PVC) (leukocytes surrounding blood vessels), mucus (basophilic wispy material within airways), and peribronchiolar cuffing (leukocytes surrounding bronchioles), using a scale from 0 (absence of pathology) to 4 (severe pathology). For each mouse, scores for all parameters were added up to generate an individual total pathology score. An average group pathology score was then calculated as the mean of individual total scores (*n* = 5 per group). Statistical significance between groups was assessed by one-way ANOVA followed by Tukey’s multiple comparison test.

### 2.8. ELISA

To measure serum IgG, His-tagged purified preF (4 µg/mL in PBS), postF (1 µg/mL in PBS), or G (4 µg/mL in PBS) were incubated on 96-well plates overnight. Purified preF and G proteins were acquired from Sino Biological. PostF protein was kindly provided by Jason McLellan (University of Texas at Austin, Austin, TX, USA). Antigen-coated plates were washed, blocked, and incubated with mouse sera (pooled from 5 mice/group) diluted in 3-fold steps, with a starting concentration of 1:100. Primary Abs were incubated for two hours at room temperature, followed by two wash steps. HRP-conjugated secondary Ab was incubated for one hour. Plates were washed three times and developed using O-phenylenediamine dihydrochloride substrate (Fisher Thermo Scientific, Waltham, MA, USA) as above. Ab titration curves were analyzed using GraphPad Prism10 software (San Diego, CA, USA), and the area under the curve (AUC) was calculated. Statistical differences were determined by one-way ANOVA with Tukey’s post hoc test. Data are presented as mean ± SD.

To measure lung IgA, ELISA and quantitation were similarly performed with minor differences. Ab levels were determined for individual mice (*n* = 5/group). Lung homogenates were serially diluted two-fold starting with a 1:2 dilution, and the secondary HRP-conjugated Ab was specific for IgA (Southern Biotech, Birmingham, AL, USA). Data represent the mean ± SD of five individual mice.

### 2.9. In Vitro Neutralization

A recombinant virus that expresses horseradish peroxidase (RSV-HRP) was previously generated [20] and used for neutralization assays. Serum samples from each mouse group (*n* = 5) were pooled and heat-inactivated at 56 °C for 30 min. No exogenous complement was added, ensuring the neutralization is specific to complement-independent Ab neutralization. RSV-HRP (300 PFU per sample) was mixed with 3-fold serially diluted serum samples. After a 1-h incubation, the mixtures were transferred to freshly plated HEp2 cells, incubated for 1 h, and then replaced with fresh media. After 48 h of incubation at 37 °C, HRP expression was measured by ELISA by replacing the well supernatants with O-phenylenediamine dihydrochloride substrate and reading OD_490_ 30 min later. The dilutions that resulted in 50% neutralization were determined using non-linear curve fits (GraphPad Prism 10 (San Diego, CA, USA)). Sera from mock-vaccinated mice and random mice IgGs were used as negative controls. The experiment was carried out twice with similar results.

### 2.10. Statistical Analyses

All statistical analyses were performed using GraphPad Prism 10 software (San Diego, CA, USA). The data are represented as the mean ± SD unless otherwise stated. *p*-values < 0.05 were considered statistically significant. Statistical tests are indicated in the figure legends, and the statistical significance is denoted as * *p* < 0.05, ** *p* < 0.01, *** *p* < 0.001, and **** *p* < 0.0001. In vitro experiments were carried out at least twice with similar results.

### 2.11. Ethics Statement for Animal Work

All mouse studies were approved by the Institutional Biosafety Committee and Institutional Animal Care and Use Committee at Oklahoma State University (OSU Stillwater animal assurance number A3722-01; approval protocol: IACUC-20-04, approval date: 23 January 2020; approval protocol: IACUC-20-04, approval date: 14 March 2023; approval protocol: IACUC-26-14, approval date: 25 March 2026). Female BALB/c mice were purchased from Jackson Laboratory (Bar Harbor, ME, USA) or Charles River (Wilmington, MA, USA). Experiments were conducted according to the guidelines of the Office of Laboratory Animal Welfare and the Public Health Service Policy on Humane Care and Use of Laboratory Animals. Animals were euthanized as per the guidelines of the American Veterinary Medical Association.

### 2.12. Generative Artificial Intelligence

No generative AI was used in this paper.

## 3. Results

### 3.1. Schematic Overview of Protein and Virus Constructs and Generation of Single-Cycle RSV Vaccines

**Figure 1:**
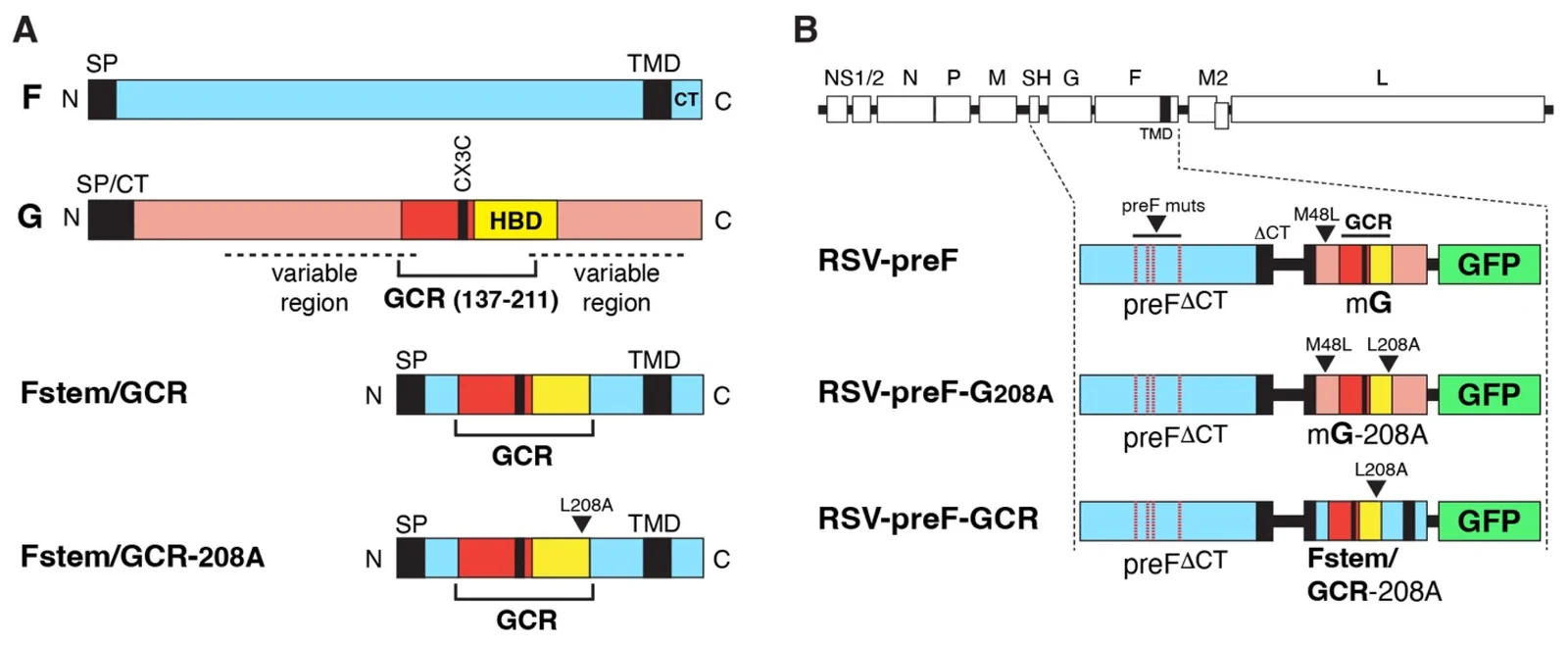
Schematic overview of single-cycle RSV viruses. (**A**) Generation of Fstem/GCR-208A. Previously reported Fstem/GCR [37], which is a G-F hybrid protein containing G residues 137–211, was modified by mutating L208 to alanine (L208A). The latter prevents premature cathepsin L cleavage in (Vero-derived) production Vbac cells. SP = signal peptide; TMD = transmembrane domain; CT = cytoplasmic tail; CX3C = RBD; HBD = heparin-binding domain, not to scale [26,27]. (**B**) The Fstem/GCR-208A ORF was used to replace the mG ORF in RSV-preF, and the new construct was named RSV-preF-GCR. As a comparison, the full G ORF was also modified with L208A and used to replace mG in RSV-preF. This construct was named RSV-preF-G208A.

Previous single-cycle vaccines were effective in inducing broad immunity and protecting animals from challenge. However, these vaccines included the full G protein (298 amino acids), which has two large, highly variable domains that can induce strain-specific rather than cross-protective anti-G Abs [47]. In contrast, the central region of G is relatively conserved and moreover contains an important RBD called CX3C. The goal of this project was to focus the anti-G immune response on the central region and thereby enhance the potential for cross-reactivity and vaccine efficacy and safety. To that purpose, we used a previously reported F/G hybrid protein termed Fstem/GCR [37]. Fstem/GCR lacks the F ectodomain (residues 36–495) and contains instead G residues 137–211, which include the central conserved region (Figure 1A). This G sequence also includes the CX3C RBD and a portion of the HBD [25,26,27]. We demonstrated that Fstem/GCR was efficiently expressed and recognized by Abs L9 [43] and 131-2G [48], which are anti-G Abs known to recognize the central region of G [37]. Because in Vero cells G residue L208 is cleaved by cathepsin L (CatL), which impacts its ability to enter primary airway cells, as a precaution we mutated L208 to alanine; L208A was shown to block cleavage and did not appear to impact G function [27,49]. The resulting protein was termed Fstem/GCR-208A. To generate a single-cycle preF-expressing virus that induces immunity only against the central region of G, a previously reported virus, RSV-preF^∆CT^ [20] (here termed RSV-preF), was modified to incorporate the ORF for Fstem/GCR-208A. RSV-preF expresses the DS-Cav1 variant of F and has an M48L mutation in the G ORF, which blocks expression of the virulence factor secreted G (sG) and thus solely expresses the membrane-bound G protein (mG). Using the remote-cutting enzyme BsmBI and conventional cloning techniques, the mG ORF from RSV-preF was replaced with that of Fstem/GCR-208A, and the resulting virus was named RSV-preF-GCR. To characterize the vaccine potential of RSV-preF-GCR, we compared it to RSV-preF-G208A, which expresses the full mG protein but is otherwise identical.

### 3.2. Viral Protein Expression in Infected Cells and Protein Composition of Virions

**Figure 2:**
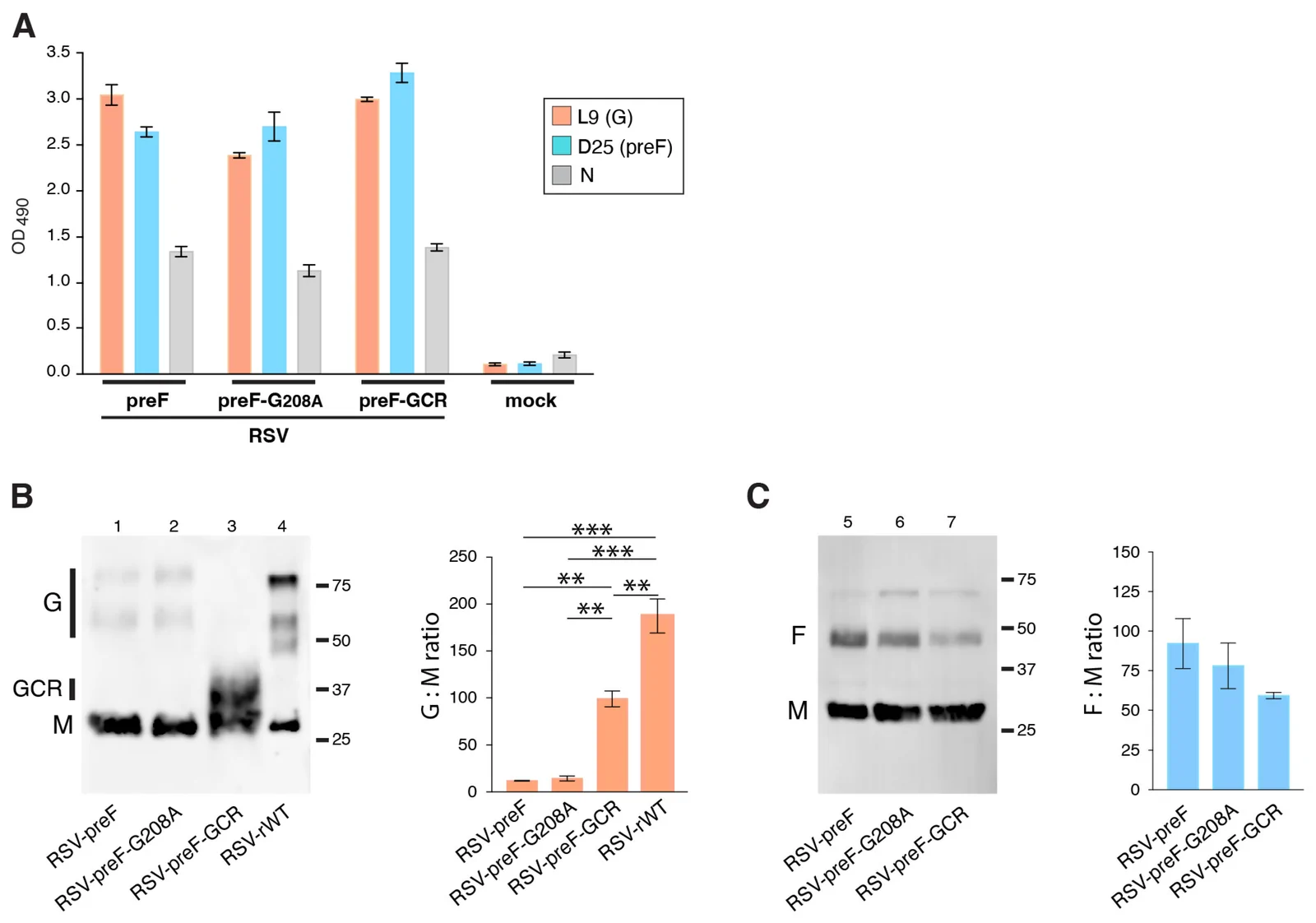
F and G protein expression in infected cells and virion protein composition. (**A**) Surface expression of preF and G proteins in infected cells. HEp-2 cells were infected with the viruses indicated, or mock-infected. To maintain native F and G conformation, unfixed cells were incubated with anti-G and anti-preF Abs, and surface levels were determined by cell ELISA. To detect N protein levels (as an indicator of equal infection rates), cells were permeabilized prior to incubation with Abs. (**B**,**C**) Protein content of vaccine viruses: G protein (**A**); F protein (**C**). Semi-purified virus stocks were pelleted at high g-force, and virion F and G levels were analyzed by Western blot. (**A**–**C**) Error bars represent standard deviations of the mean, and the experiments were carried out at least 2× independently. Only significant differences are indicated (** *p* < 0.01; *** *p* < 0.001). Note that the results of RSV-preF, RSV-preF-G208A, and mock are shown for comparisons and controls, respectively, and were previously published in Lamichhane et al., 2022 [21].

We and others have previously established that L208A does not have unintended deleterious effects on viral replication or viral protein expression [21,27]. F and G surface expression levels were examined in cells infected by the single-cycle viruses. After infecting HEp-2 cells at 2 PFU/cell, cells were incubated with anti-G Ab L9 or anti-preF Ab D25 [50], as previously described [20,21,37] (Figure 2A). An anti-N Ab was used to verify equal infection rates between the different viruses. All Abs, except N, were incubated on unfixed cells to maintain the native conformation of F and G proteins. The results show that virus RSV-preF-GCR induced levels of preF and G proteins at the cell surface similar to the levels of RSV-preF and RSV-preF-G208A.

We also examined the G and F protein content of virions by Western blot (Figure 2B,C). Equal amounts of semi-purified virus stocks were pelleted at high g-force and electrophoresed on denaturing gels. For G, as a comparison, RSV-rWT was included. After transfer, the G proteins on the blot were detected using anti-G Ab L9 (Figure 2B). F and M were detected respectively by Motavizumab and a previously described anti-M peptide serum (Figure 2C) [20,45]. As observed before [21], RSV-preF virions contained very low levels of G protein relative to RSV-rWT, and the same was true for RSV-preF-G208A. As expected, in contrast to RSV-preF and RSV-preF-G208A, RSV-preF-GCR virions lacked the full-size G protein and displayed only the Fstem/GCR-208A protein. Levels of Fstem/GCR-208A protein in virions were significantly higher than levels of full G protein in RSV-preF and RSV-preF-G208A virions. All three viruses contained similar levels of F protein.

### 3.3. RSV-preF-G208A and RSV-preF-GCR Do Not Spread to Neighboring Cells

**Figure 3:**
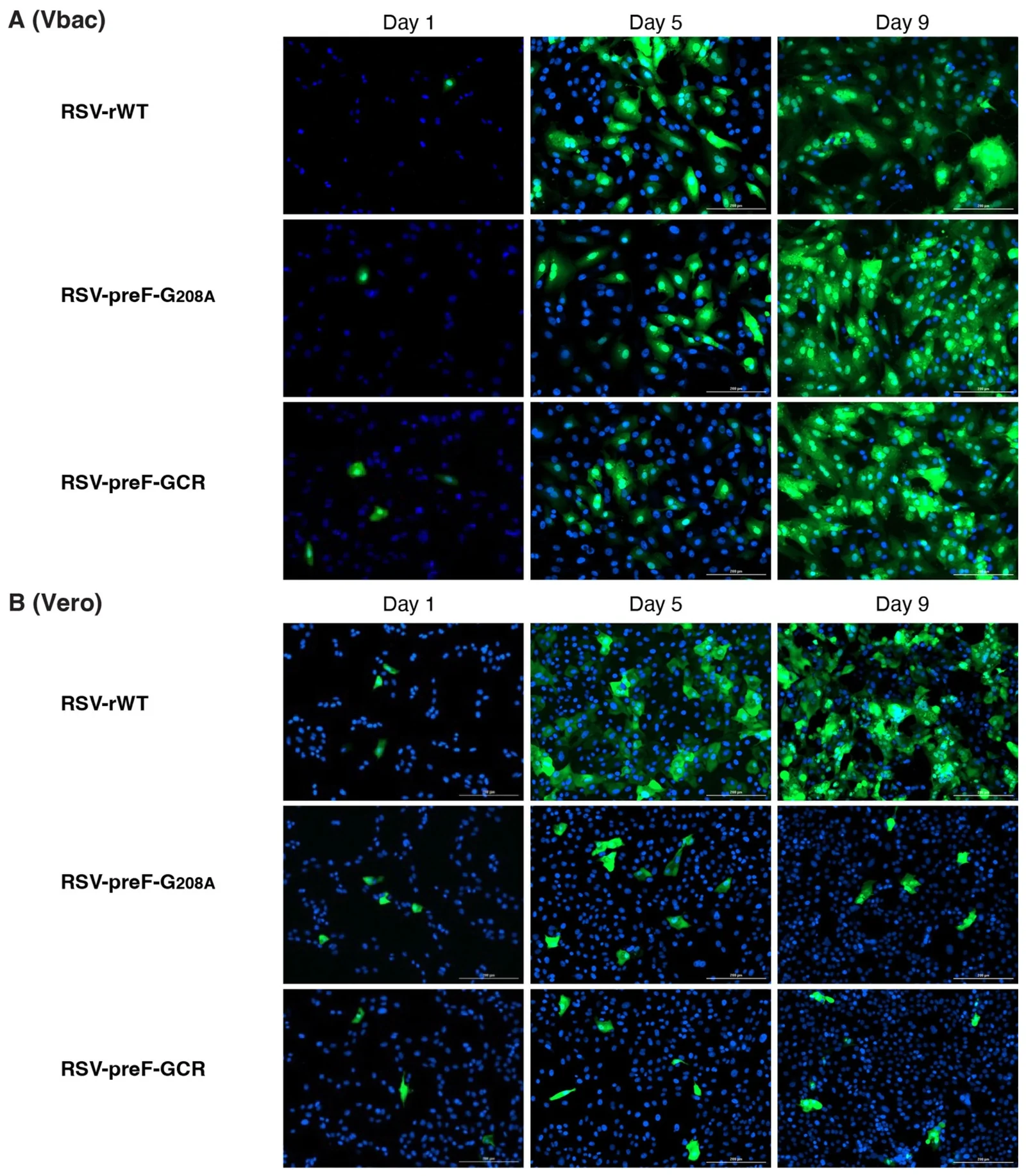
Single-cycle viruses do not transmit between cells. RSV-rWT, RSV-preF-G208A, and RSV-preF-GCR were used to infect (**A**) Vbac cells (Vero-derived production line expressing baculovirus GP^64/F^), or (**B**) Vero cells, at 0.01 PFU/cell. Cells were incubated at 33 °C for nine days, monitored for GFP expression, and fluorescent images were acquired on days 1, 5, and 9 post-infection (scale bars 200 µm) on a BioTek Cytation 5 (Agilent, Winooski, VT, USA), except for the images of Vbac, Day 1, which were taken on a Nikon TE2000 fluorescent microscope (Nikon Instruments Inc., Melville, NY, USA), 100× magnification. Nuclei were counterstained with Hoechst dye. Representative images are shown.

We previously showed that parental RSV-preF did not spread from cell to cell in Vero cell cultures [20]. To demonstrate cell–cell transmission deficiency of single-cycle viruses RSV-preF-G208A and RSV-preF-GCR, Vbac and Vero cells were infected with 0.01 PFU/cell. As a comparison, cells were similarly infected with RSV-rWT. Infected cells were incubated at 33 °C because GP64 complementation is more efficient at reduced temperature and stocks are typically grown at 33 °C [51]. Following incubation, GFP expression was monitored for 9 days. Images of day 1, 5, and 9 post-infection are shown in Figure 3. In Vbac cells (the production line), all viruses showed evidence of cell–cell transmission, resulting in a completely infected monolayer by day 9 (Figure 3A). RSV-rWT also spread efficiently in Vero cells (Figure 3B). In contrast, RSV-preF-G208A and RSV-preF-GCR did not spread beyond the initially infected Vero cells. Even by day 9 pi, only a few green cells were present in wells infected by RSV-preF-G208A and RSV-preF-GCR, and uninfected cells kept dividing as evidenced by the large number of Hoechst-stained nuclei.

### 3.4. Antibody Induction

**Figure 4:**
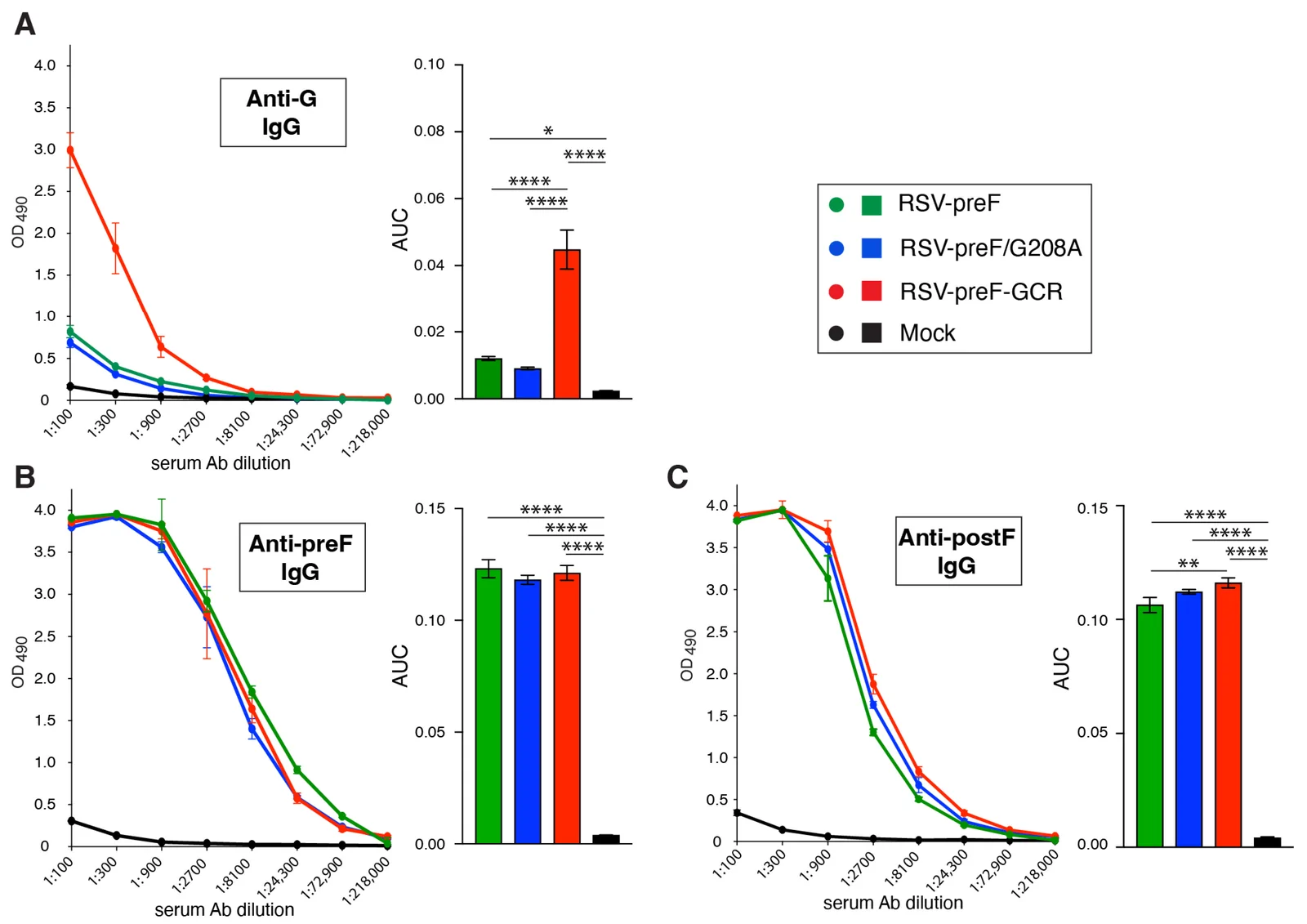
Serum levels of IgG Abs after prime-boost vaccination. Eight-week-old BALB/c mice were IN vaccinated 2× with RSV-preF, RSV-preF/G208A, or RSV-preF-GCR three weeks apart with 0.5 million PFU/animal. Mock vaccine was generated exactly as the experimental vaccines but from uninfected Vbac cells. Blood samples were taken three weeks post-boost. 3-Fold dilutions of pooled boost sera from mice (total *n* = 5/group) were incubated on plates coated with purified G (**A**), preF (**B**), or postF (**C**) proteins. Ab curves were analyzed using GraphPad Prism to determine the AUC and compared using one-way ANOVA. Error bars represent the standard deviation of the mean of triplicate samples. Only statistically significant differences are indicated (* *p* < 0.05; ** *p* < 0.01; **** *p* < 0.0001). The ELISAs were carried out twice with similar results.

To examine the ability of RSV-preF-GCR to induce anti-G and anti-preF Abs, RSV-preF-GCR was used to vaccinate mice. This was done according to a previously established protocol in which eight-week-old BALB/c mice received a prime and boost IN vaccination of 0.5 million PFU, 3 weeks apart. A mock-vaccinated group was included as a negative control, for which mock vaccine was prepared identically from uninfected Vbac cells. Serum Abs were harvested 3 weeks post-boost and examined by ELISA for Ab levels (Figure 4). In addition to anti-G Abs (Figure 4A), we also measured anti-F Ab levels (Figure 4B,C) to ensure that the G modifications would not negatively impact anti-F Abs. To identify anti-F and anti-G Abs, the ELISA used purified His-tagged G, preF, and postF antigens as targets, as previously described and validated [20]. To limit animal numbers, we did not include RSV-rWT vaccination, which we previously reported on [20,37]. Upon examination of vaccinated mouse sera and quantitation of Ab levels, RSV-preF-GCR showed robust anti-preF and anti-postF Abs at levels similar to the other vaccines. With regard to anti-G Abs, RSV-preF induced very low levels, which we also observed in previous work [20,21]. RSV-preF-GCR, however, shows substantially higher anti-G Ab levels, though levels appeared moderate compared to levels induced by RSV-rWT in previous work [20,37]. Thus, in contrast to RSV-preF, RSV-preF-GCR induced readily detectable levels of both anti-preF and anti-G Abs.

### 3.5. RSV-preF-GCR-Induced Anti-G Abs Are Specific for the GCR

**Figure 5:**
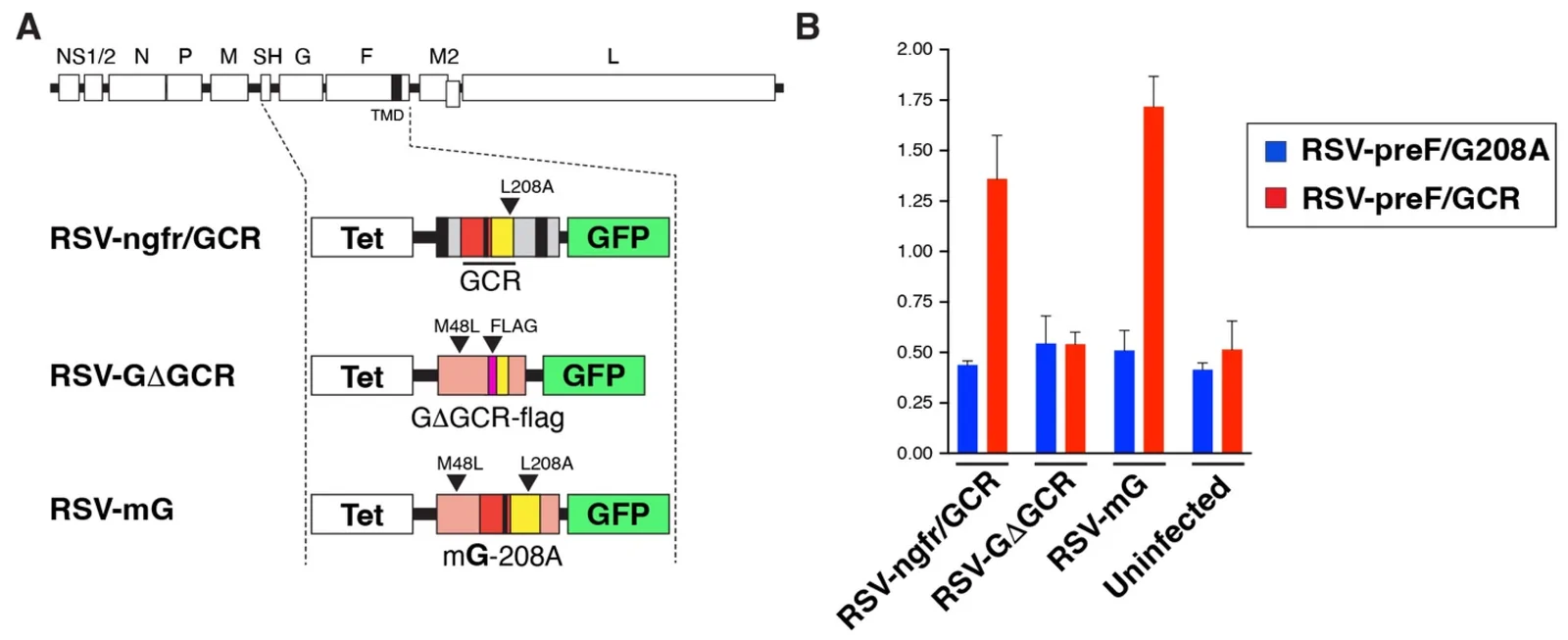
Induced serum anti-G Abs are specific for the GCR. (**A**) Three diagnostic viruses were constructed to demonstrate GCR-specificity. These viruses differed only in G protein content, one expressing the G protein GCR grafted onto NGFR for strong surface expression (RSV-ngfr/GCR), another expressing a G protein in which the GCR was replaced with a flag tag (RSV-GDGCR), and a third expressing a wild-type membrane-anchored G protein (RSV-mG). (**B**) Demonstration of GCR-specificity. HEp-2 cells were infected with the three diagnostic viruses or left uninfected as a negative control. At 24 hpi, sera from mice vaccinated with RSV-preF-GCR or RSV-preF-G208A were incubated on the infected HEp-2 cells, and the level of anti-G Abs was determined by cell ELISA. Error bars represent the standard deviation of the mean of triplicate samples from pooled sera.

RSV-preF-GCR was designed to induce Abs against the relatively conserved central region by lacking a large portion of the amino- and carboxy-terminal variable regions of the G ORF (amino acids 1 to 136 and 212–298, respectively) in the viral genome. To verify that the anti-G Abs, as observed in Figure 4, were specific for the GCR, we generated three viruses for diagnostic purposes, termed RSV-ngfr/GCR, RSV-G∆GCR, and RSV-mG (Figure 5A). All three lack the SH and F ORFs, and express either a full-length or modified version of the G protein. For RSV-ngfr/GCR, the GCR was cloned into the ORF of nerve growth factor receptor from which the ectodomain was removed. This virus thus expresses only the GCR and contains no other RSV glycoprotein coding sequences. In RSV-mG, the methionine at position 48 was ablated to express only the membrane-anchored form of G (mG). For RSV-G∆GCR, the GCR was deleted from the G protein and replaced with a flag tag (G∆GCR-flag). To verify surface expression of G∆GCR-flag, cells were infected with RSV-G∆GCR and compared to a virus expressing wt G protein (RSV-G) using anti-FLAG Ab and anti-G Ab L9 (Figure S1). This experiment showed that G∆GCR-flag is abundantly expressed at the cell surface. To demonstrate GCR-specificity for Abs induced in RSV-preF-GCR vaccinated mice, HEp-2 cells were infected with RSV-ngfr/GCR, RSV-G∆GCR, RSV-mG (as a comparison), or left uninfected (Figure 5B). At 24 h post-infection (hpi), Abs from mice vaccinated with RSV-preF-GCR and RSV-preF-G208A were incubated with the infected cells, and relative levels of G Abs were determined by ELISA.

Abs derived from mice vaccinated with RSV-preF-GCR yielded a signal above background in cells infected with RSV-ngfr/GCR but not in cells infected with RSV-G∆GCR, showing that these Abs were specific to the GCR. In sera from RSV-preF-G208A vaccinated mice, no anti-G Abs were detected. This is in agreement with Figure 4 and with our previous work, in which we found that a preF-based single-cycle virus containing a wt G gene in its native position induces surprisingly low anti-G Ab levels [20,21].

### 3.6. Virus Neutralization

**Figure 6:**
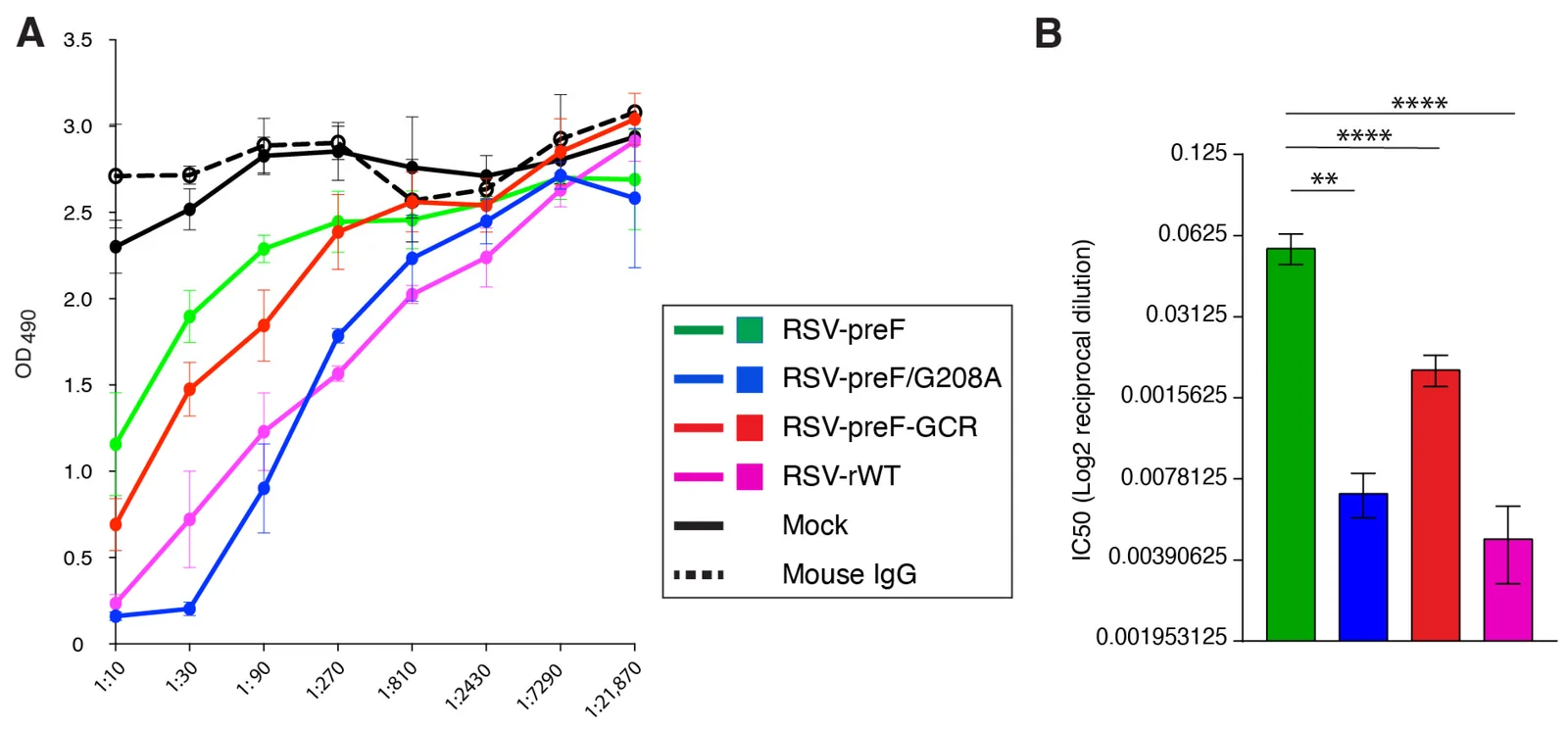
In vitro virus neutralization. (**A**) Neutralization capacities of heat-inactivated mouse sera were determined using wt RSV engineered to express horse-radish peroxidase (RSV-HRP) as described previously [20]. Random mouse IgG and sera from mock-vaccinated mice were included as negative controls. The data points represent the mean of triplicate samples of pooled sera from 5 mice/group. (**B**) Non-linear curve-fit analyses were performed using Prism10, to determine statistical differences. Only statistically significant differences are indicated. Error bars are the standard deviation of the mean (** *p* < 0.01; **** *p* < 0.0001). The experiment was performed twice with similar results. Note that the results of RSV-preF, RSV-rWT, mock, and mouse IgG are shown for comparisons and controls, respectively, and were previously published in Lamichhane et al. 2022 [21].

To examine neutralization potential, we performed an in vitro neutralization assay (Figure 6). As a readout, we used a previously described and validated virus expressing HRP (RSV-HRP) [20,21]. Pooled sera from vaccinated mice were heat-inactivated, incubated with RSV-HRP (without added complement), and then used to infect HEp-2 cells. At 48 hpi, medium was replaced with ELISA substrate and the OD_490_ was determined. In spite of higher anti-G Ab levels as observed in Figure 4, RSV-preF-GCR-induced Abs did not show improved neutralization relative to RSV-preF-G208A. In fact, RSV-preF-G208A-induced Abs significantly outperformed RSV-preF-GCR and neutralized as well as Abs from RSV-rWT-vaccinated mice. Interestingly, RSV-preF-G208A-induced Abs also significantly outperformed RSV-preF.

This suggests that the G protein is cleaved by Cathepsin L in Vbac production cells, that the cleavage is prevented by the L208A mutation [27], and that the cathepsin L cleavage negatively impacts induction of neutralizing anti-G Abs. RSV-preF-GCR neutralized moderately better than RSV-preF. Since the level of anti-G Abs is the only observed difference between the two viruses, this suggests that the improved neutralization is due to the anti-GCR Abs.

### 3.7. Induction of Lung IgA

**Figure 7:**
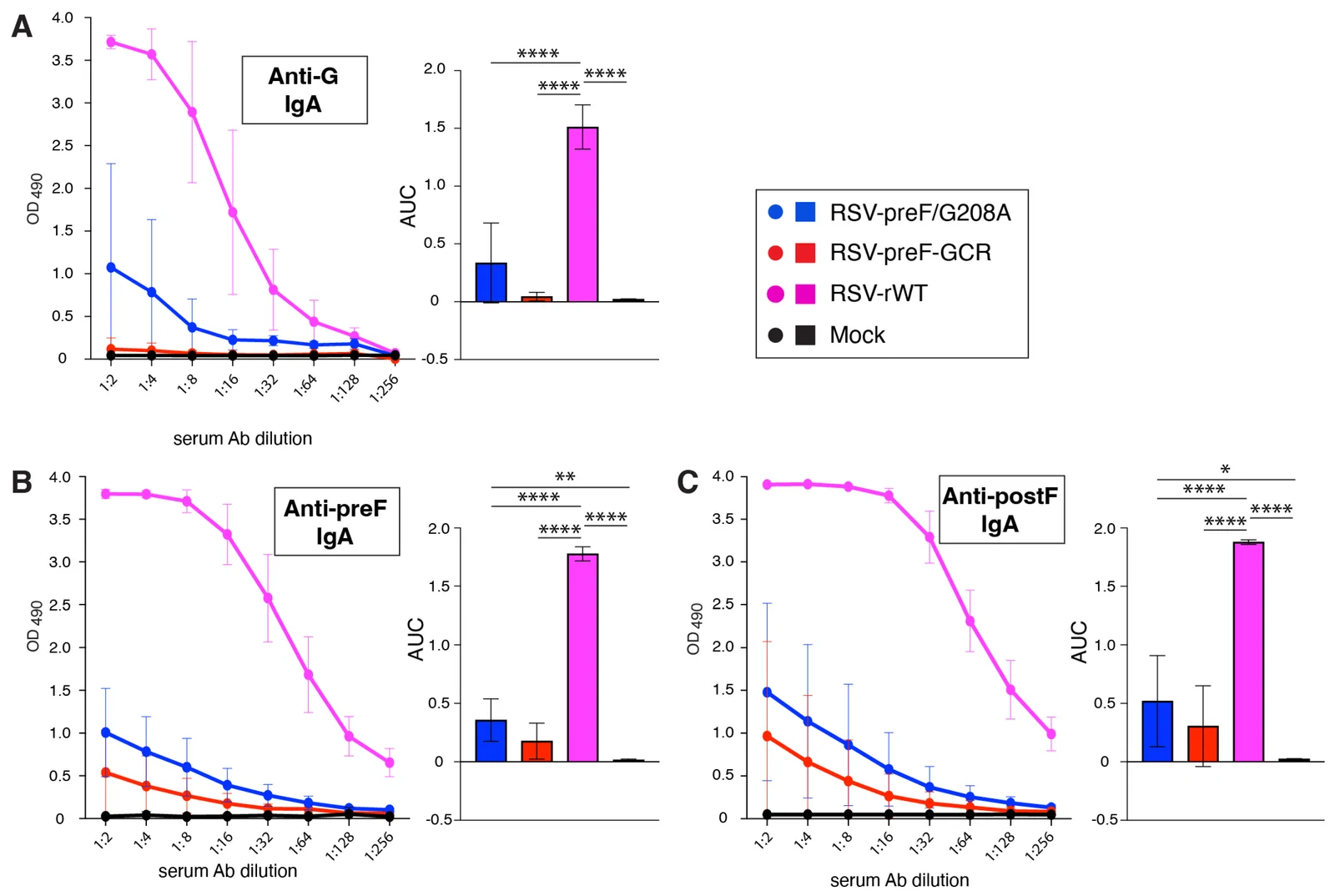
Lung IgA levels two weeks after prime-boost vaccination. Eight-week-old BALB/c mice were twice IN vaccinated with RSV-preF, RSV-preF/G208A, or RSV-preF-GCR three weeks apart with 0.5 million PFU/animal. Mock vaccine is uninfected Vbac cells prepared in parallel with the vaccines. RSV-rWT was included as a positive control. Lungs were collected two weeks post-boost, homogenized, and stored at −80 °C after clearing cell debris. 2-Fold dilutions of lung homogenates from mice (*n* = 5/group) were incubated on plates coated with purified G (**A**), preF (**B**), or postF (**C**) proteins. Ab curves were analyzed using GraphPad Prism, quantitated by AUC, and compared using one-way ANOVA. Error bars represent the standard deviation of the mean. Only statistically significant differences are indicated (* *p* < 0.05; ** *p* < 0.01; **** *p* < 0.0001).

As IN-applied vaccines have the potential to induce mucosal immunity, we examined whether viruses RSV-preF-G208A and RSV-preF-GCR induced lung IgA in addition to serum IgG (Figure 7). Eight-week-old BALB/c mice (*n* = 5/group) received a prime-boost vaccination as described above. RSV-rWT was included as a control. Animals were euthanized two weeks post-boost, and lungs were perfused with PBS and homogenized.

Cleared homogenates were stored at −80 °C and examined for Ab levels by ELISA, again using purified G (Figure 7A) and F (Figure 7B,C) antigens. A conjugated secondary Ab specific for the IgA isotype was used to detect IgA Abs. Dilution curves were generated, and responses were quantitated by AUC. We found that both RSV-preF-G208A and RSV-preF-GCR induced very low levels of IgA in the lung, relative to RSV-rWT. RSV-preF-G208A induced low but significant amounts of anti-preF and anti-postF IgA, whereas RSV-preF-GCR did not induce significant levels of anti-G or F IgA. Thus, in contrast to a wild-type virus, and in spite of IN application, the single-cycle viruses were poor inducers of lung IgA.

### 3.8. Protection from Challenge

**Figure 8:**
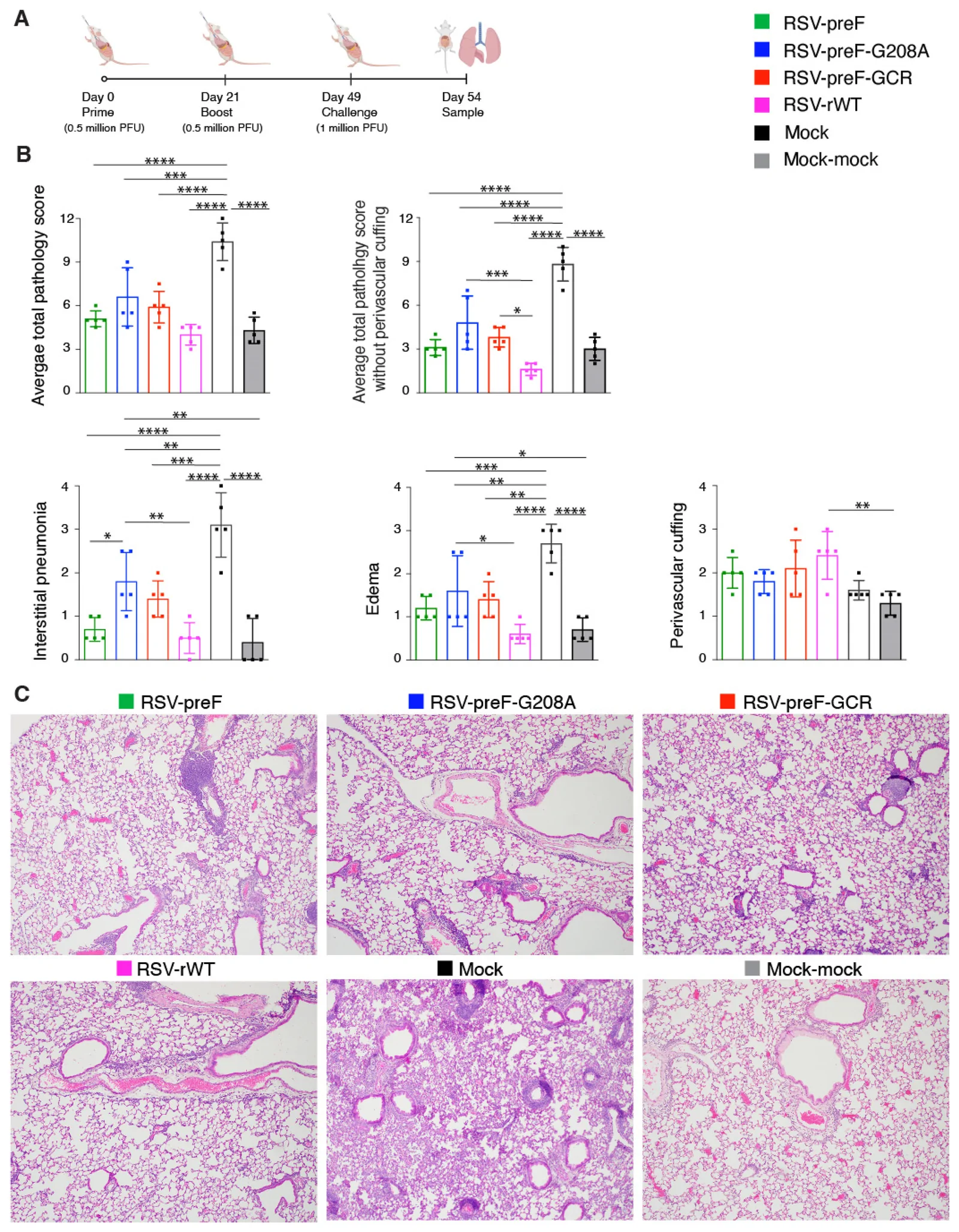
Protection from lung pathology after challenge with wt RSV. Mice were vaccinated prime-boost and challenged with RSV (RSV-K191R [21]) at 4 weeks post-boost. Five days post-challenge, lungs from challenged mice were fixed, processed for histological examination, and scored blindly by a certified veterinary pathologist. (**A**) Schematic overview of the protocol. (**B**) Average total pathology scores (all parameters combined) with or without PVC and average individual parameter scores. Error bars represent the standard deviation of the mean. Statistical significance between groups was assessed by one-way ANOVA followed by Tukey’s multiple comparison test. (* *p* < 0.05; ** *p* < 0.01; *** *p* < 0.001; **** *p* < 0.0001; only statistically significant differences are indicated). (**C**) Images of H&E-stained lung sections, representative of the scores in panel (**B**). Magnification, 100×.

To determine whether the response to vaccination could protect from lung pathology, mice were vaccinated using a prime/boost regimen as above and challenged with 1 million PFU of wild-type RSV. The RSV-K191R variant [20] was used, which carries an RSV line19F mutation [52] and induces more weight loss in BALB/c mice than its parent RSV A2 strain [21]. Mock-vaccinated mice were included as a negative control for protection, in which mock was derived from uninfected Vbac cells and processed identically. In addition, a group was included that received two mock vaccinations and a mock challenge (Mock-mock), and this group thus never encountered virus. Five days post-challenge, lungs were harvested and processed for histological examination, using H&E stain (see Section 2) (Figure 8). Tissues were examined and scored blindly by an ACVP board-certified veterinary anatomic pathologist for five parameters commonly assessed for RSV-induced pathology, scoring each parameter from 0 (no pathology) to 4 (high pathology): interstitial pneumonia (increased thickness of alveolar septae by increased number of leucocytes), edema (extrusion of amorphous eosinophilic material into the airways and surrounding blood vessels, and/or presence of foamy macrophages), PVC (leukocytes surrounding blood vessels), peribronchiolar cuffing (leucocytes surrounding bronchioles), and mucus (basophilic whispy material within airways). Peribronchiolar cuffing and mucus were not detected. As observed in previous work, PVC was detected evenly across all groups [20,21,37], and an average total pathology score for each group was determined with and without PVC.

As expected, mock-vaccinated mice had the highest average total pathology scores, which were much higher than those from the Mock-mock group. The latter had consistently low scores and showed no signs of pathology in the H&E-stained sections. All vaccines showed significant protection from challenge. Except for PVC as a parameter, RSV-rWT outperformed all single-cycle vaccines and also had the lowest average total pathology score. Although RSV-preF-G208A showed higher neutralizing titers than RSV-preF and RSV-preF-GCR in Figure 6, there were no significant differences between RSV-preF-G208A and RSV-preF-GCR in protection against pathology. Moreover, RSV-preF vaccination moderately outperformed RSV-preF-G208A. Overall, the single-cycle viruses significantly lowered the pathology scores but did not protect from lung pathology as well as RSV-rWT, with RSV-preF-G208A vaccination inducing the weakest protection against challenge. This was in spite of performing better than RSV-preF and RSV-preF-GCR in in vitro neutralization.

## 4. Discussion

### 4.1. Rationale for a GCR-Focused Immune Response

Based on a body of published work that points to the central G protein region as an important vaccine target (see Section 1), we here generated a single-cycle preF-based vaccine expressing G amino acids 137–211. When residues 137–211 were grafted onto Fstem, the resulting hybrid construct (Fstem/GCR) was expressed abundantly at the surface of infected cells [37]. In this project, we used Fstem/GCR to replace the G ORF in a single-cycle vaccine background, in an effort to direct immunity towards the central region of G. As explained in the Section 1, there are several potential advantages. Chief among those are the presence of an RBD within the GCR and the higher conservation of the GCR, relative to the GCR flanking regions. Any neutralizing Abs targeted to the highly variable flanking regions would likely still help protect against the same (homologous) strain. However, such Abs would likely not protect against different RSV strains [47] due to the extreme variability of the flanking regions. Thus, Abs targeted to the G flanking regions would negatively impact efficacy in case of diverse strains and could also contribute to more severe disease [40,41]. It has also been suggested that the conserved region is poorly immunogenic in the context of full-length G protein [47]. Altogether, there is a strong case for an anti-G response targeted exclusively to the conserved region.

### 4.2. Serum Anti-G Ab Induction

An RSV-preF-GCR batch was readily generated in the Vbac production line and was characterized in vitro and compared in vivo to an identical virus that expresses a full-length G (RSV-preF-G208A). RSV-preF-GCR induced anti-G Abs at levels significantly higher than RSV-preF-G208A (Figure 4). However, these Ab levels were not as high as Ab levels seen in our previous work with a wild-type RSV [20,21]. We also reported previously that RSV-preF, which expresses a full-length G (without the L208A mutation) from the native (7th) genome location, induced only barely detectable Ab levels [20,21]. This was unexpected, as the virus expresses high levels of G at the cell surface in infected cells. The virus RSV-preF-G208A matched our previous RSV-preF findings in also inducing very low anti-G Ab levels in mice. In that regard, it was surprising that RSV-preF-GCR induced higher anti-G Ab levels in vivo, because Fstem/GCR is expressed from the same native G location. In the case of RSV-preF, moving G to the first genome position led to better virion incorporation and enhanced anti-G Ab levels [21]. In the case of RSV-Fstem-GCR, we also found better incorporation of Fstem/GCR into virions and enhanced anti-G Ab levels. The latter cannot be attributed to genome location, as Fstem/GCR is expressed from the native G location. Rather, a possible explanation for higher anti-G Ab levels is strong Fstem/GCR virion incorporation in the presence of the F protein CT, which we and others previously showed to facilitate viral assembly and virion incorporation [37,44,46,53]. Combined, the data suggest that G incorporation into the virion can be achieved in different ways and ultimately leads to higher anti-G Ab levels, be it via direct input G antigen processing or via improved virus binding/entry or immune cell uptake due to higher G levels.

### 4.3. Neutralization Potential

In humans, we would expect Abs induced by RSV-preF-GCR to improve G protein-based neutralization relative to RSV-preF-G208A because (1) it contains higher levels of G (Fstem/GCR) antigen in the virion (Figure 2); (2) it induces higher anti-G Ab levels than RSV-preF-G208A (Figure 4); (3) the induced Abs are directed towards the more conserved region of G (Figure 5), which harbors an important RBD (CX3C) [28,29]. In addition, anti-GCR Abs are likely to better cross-protect against different strains due to higher conservation relative to the flanking regions. In Figure 6, however, we found that RSV-preF-GCR-induced Abs neutralized poorly compared to those induced by RSV-preF-G208A. This could potentially be explained by the fact that, in cultured cell lines, RSV also enters in a non-specific manner using its HBD [25,26,27,28,29]. Thus, it is possible that Abs induced by RSV-preF-GCR vaccinated mice indeed effectively bind the G protein central region, but do not block in vitro viral entry mediated by G’s linear HBD. This limitation of the in vitro assay, as well as lower relative anti-G Ab levels, may explain why RSV-preF-GCR-induced Abs do not neutralize as well as RSV-rWT. For this reason, we are currently developing a neutralization assay based on primary cells such as normal human bronchial epithelial cells to avoid potential interference from the HBD. Simultaneously, we plan to modify the vaccine to enhance anti-GCR Ab levels. With regard to the neutralization results, it is also possible that, in the case of RSV-preF-G208A, Abs against the flanking regions contributed to neutralization, since the strain used for neutralization is the same (A2) as the candidate vaccines.

The role of the G mutation L208A. It is not clear whether mutation L208A improves neutralization or vaccine potential. For RSV-preF-GCR, Cathepsin L cleavage of L208 would truncate the Fstem/GCR by only a few amino acids, which may not have an impact. For full-length G, however, mutation L208 did have an impact: When comparing RSV-Fstem-G208A and RSV-preF, which differ only in one amino acid (G residue 208), we found that RSV-Fstem-G208A-induced Abs neutralized significantly better. This suggests that L208A indeed prevents Cathepsin L cleavage in Vbac cells and that the additional neutralization is directed to the C-terminal variable domain. Neutralizing Abs against the C-terminal variable region have been described before and have even been suggested to be immuno-dominant [47], which may underlie the variability of this region and lead to low anti-GCR Ab levels. Although successful against a homologous RSV strain as in Figure 6, Abs directed at the C-terminus would likely be strain-specific and poorly cross-protective.

Protection against challenge. Since RSV-preF-GCR induced higher anti-G Ab levels than RSV-preF-G208A, we expected the former to protect better against challenge-related lung pathology. However, experimental vaccines RSV-preF-G208A and RSV-preF-GCR reduced lung pathology to a similar extent, whereas RSV-rWT vaccination slightly outperformed the single-cycle viruses. Thus, having significant levels of GCR-focused Abs did not improve protection relative to RSV-preF-G208A, which induced very low levels of anti-G Abs. Studies in human airway epithelial cells indicate that CX3C-CX3CR1 interaction is important for viral entry in vivo [28,29], which in turn would predict that anti-GCR Abs reduce viral infection. At this time, we do not know why RSV-preF-GCR failed to improve protection. If IgA plays an important role in preventing infection and/or pathology, then the low levels of IgA induced by the single-cycle viruses might explain why RSV-rWT affords moderately better protection, as well as only minor differences between RSV-preF-GCR and RSV-preF-G208A. Another potential explanation could be related to T cells. In previous work, we showed that RSV-preF^∆CT^ induced levels of RSV-specific T cells similar to a wt RSV [20]. Although we expect the experimental vaccines to similarly induce T cells, we have not examined this in this study.

### 4.4. Lung IgA

An undesirable finding was that RSV-preF-G208A and RSV-preF-GCR induced very low levels of lung IgA compared to a wild-type RSV control, and this was true for both anti-G and anti-preF Abs. This was unexpected because of the IN route of administration of the vaccine and the fact that robust levels of serum anti-preF IgG Abs were induced. Given that the single-cycle vaccines are blocked in cell–cell transmission, one possibility is that a certain level of progeny production and concurrent cell-to-cell spreading facilitates proper induction of IgA in the lung. However, we recently showed that another single-cycle virus (based on lack of matrix protein) did induce high levels of lung IgA [22]. Moreover, downregulation of the interferon antagonist NS1 showed potential to speed up induction of lung IgA. Another possibility is that the single-cycle viruses, whose infectivity is mediated only by baculovirus GP64, enter different cell types than wt RSV or the matrix protein-based single-cycle vaccines, potentially resulting in differential replication kinetics and different antigen presentation. Alternatively, viruses may be taken up by different immune cells, thereby also potentially altering antigen presentation and the innate response. The distinct responses induced by preF-based and matrix protein-based single-cycle vaccines may present an opportunity to study the mechanical differences. For example, we could examine which cell types are infected in vivo (both epithelial and non-epithelial), and what the level of viral replication is. We could also vaccinate mice and perform single-cell RNAseq to try to identify differences in lung cell subsets between preF- and matrix protein-based vaccines. Together, single-cycle RSV viruses do appear to be capable of inducing a lung IgA response, but it is at this time not clear why RSV-preF-GCR and RSV-preF-G208A failed to do so.

### 4.5. In Summary

The in vitro neutralization assay and mouse model are critical tools to pre-clinically assess the anti-RSV immune response, but also have some limitations that make it difficult to demonstrate subtle but potentially important vaccine qualities or improvements. In addition, poor lung IgA levels induced by preF-based single-cycle viruses suggest suboptimal mucosal immunity, which may have lowered effectiveness. On the positive side, RSV-preF-GCR, a transmission-deficient single-cycle vaccine, induced GCR-specific Abs, high levels of anti-preF Abs, and significantly protected against RSV challenge, and thus has potential to improve safety, efficacy, and protection across RSV strains. The latter could be a significant advantage, and we next aim to enhance anti-GCR Ab levels and to demonstrate cross-protection against different RSV strains in future work using a heparin-independent in vitro neutralization assay. As IgA is an important component of mucosal immunity, conceivably, the differences between the preF- and M-based single-cycle viruses could be exploited to gain a better understanding of IgA induction by live RSV vaccines.

## Data Availability

The raw data supporting the conclusions of this article will be made available by the authors on request.

## Supplementary Materials

The following supporting information can be downloaded at: https://www.mdpi.com/article/10.3390/v18091013/s1, Figure S1: G protein surface expression by RSV-G∆GCR.
