# Supplementary material for: A Live Single-Cycle RSV Vaccine with Immune Responses Focused on Prefusion F and the Central Conserved Region of Attachment Protein G
Source: Viruses. 2026 Sep 14;18(9):1013. doi: 10.3390/v18091013 (PMC13612014; doi:10.3390/v18091013)
Supplement: Supplementary file 1 [file viruses-18-01013-s001.zip › viruses-4529977-supplementary.pdf]

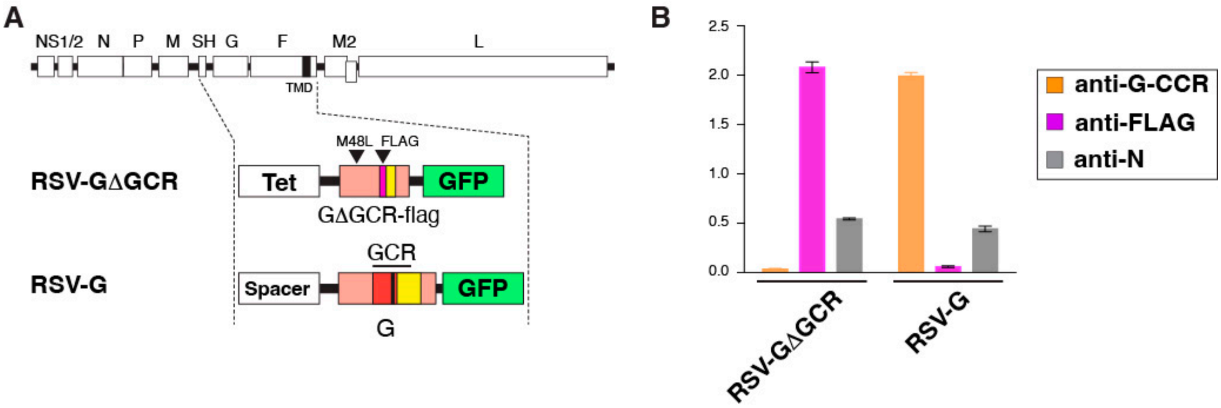

Supplementary Figure S1: G protein surface expression by RSV-GΔGCR. (**A**) schematic of diagnostic viruses RSV-GΔGCR and RSV-G, respectively expressing a G protein lacking its GCR (GΔGCR-flag) or membrane-anchored full length G protein (RSV-G). In GΔGCR-flag, the GCR (residues 137-211) were deleted and replaced with a Flag tag (purple box). Yellow boxes represent the HBD (partial for RSV-GΔGCR - amino acids 212-231). (**B**) Surface expression of G proteins. To validate surface expression of GΔGCR-flag by diagnostic virus RSV-GΔGCR, HEp-2 cells were infected with RSV-GΔGCR or RSV-G and examined by cell ELISA as previously described [20] with minor modifications. At 26 hpi, infected cells were incubated with L9, an anti-G Ab that was previously shown to bind the G-CCR [37] or anti-FLAG Ab (Genscript), prior to fixation to ensure that only surface levels are detected. Infected cells were also incubated with an Ab against the RSV N protein (B023, BioRad) which we previously validated [21] as a control for virus replication levels. In the case of N, cells were fixed and permeabilized to be able to detect N intracellularly. Following incubation with HRP-conjugated secondary Abs, the relative surface levels of GΔGCR-flag and G proteins were determined. Error bars represent the standard deviation of the mean of triplicate samples from pooled sera. The results showed that GΔGCR-flag is abundantly expressed at the cell surface.
